# Gene expressions and copy numbers associated with metastatic phenotypes of uterine cervical cancer

**DOI:** 10.1186/1471-2164-7-268

**Published:** 2006-10-20

**Authors:** Heidi Lyng, Runar S Brøvig, Debbie H Svendsrud, Ruth Holm, Olav Kaalhus, Kjetil Knutstad, Halldis Oksefjell, Kolbein Sundfør, Gunnar B Kristensen, Trond Stokke

**Affiliations:** 1 Department of Radiation Biology, Health Enterprise Rikshospitalet – Radiumhospitalet, Oslo, Norway; 2 Department of Pathology, Health Enterprise Rikshospitalet – Radiumhospitalet, Oslo, Norway; 3 Department of Roentgenology, Health Enterprise Rikshospitalet – Radiumhospitalet, Oslo, Norway; 4 Department of Gynecologic Oncology, Health Enterprise Rikshospitalet – Radiumhospitalet, Oslo, Norway; 5 Department of Medical Informatics, University of Oslo, Oslo, Norway

## Abstract

**Background:**

A better understanding of the development of metastatic disease and the identification of molecular markers for cancer spread would be useful for the design of improved treatment strategies. This study was conducted to identify gene expressions associated with metastatic phenotypes of locally advanced cervical carcinomas and investigate whether gains or losses of these genes could play a role in regulation of the transcripts. Gene expressions and copy number changes were determined in primary tumors from 29 patients with and 19 without diagnosed lymph node metastases by use of cDNA and genomic microarray techniques, respectively.

**Results:**

Thirty-one genes that differed in expression between the node positive and negative tumors were identified. Expressions of eight of these genes (*MRPL11, CKS2, PDK2, MRPS23, MSN, TBX3, KLF3, LSM3*) correlated with progression free survival in univariate analysis and were therefore more strongly associated with metastatic phenotypes than the others. Immunohistochemistry data of CKS2 and MSN showed similar relationships to survival. The prognostic genes clustered into two groups, suggesting two major metastatic phenotypes. One group was associated with rapid proliferation, oxidative phosphorylation, invasiveness, and tumor size (*MRPS23, MRPL11, CKS2, LSM3, TBX3, MSN*) and another with hypoxia tolerance, anaerobic metabolism, and high lactate content (*PDK2, KLF3*). Multivariate analysis identified tumor volume and *PDK2 *expression as independent prognostic variables. Gene copy number changes of the differentially expressed genes were not frequent, but correlated with the expression level for seven genes, including *MRPS23, MSN*, and *LSM3*.

**Conclusion:**

Gene expressions associated with known metastatic phenotypes of cervical cancers were identified. Our findings may indicate molecular mechanisms underlying development of these phenotypes and be useful as markers of cancer spread. Gains or losses of the genes may be involved in development of the metastatic phenotypes in some cases, but other mechanisms for transcriptional regulation are probably important in the majority of tumors.

## Background

Lymph node involvement is the first indication of spreading and a strong prognostic factor for epithelial cancers [1]. A better understanding of the development of metastatic tumor phenotypes and the identification of molecular markers for lymphatic spread would be useful in design of improved treatment strategies [2]. Cervical carcinomas have been studied extensively during the last years in the search for biological characteristics that are associated with lymph node involvement. Tumor volume is among the strongest prognostic factors. Severe hypoxia, high level of lactate, high proliferation rate, increased angiogenesis, high interstitial fluid pressure, and low apoptotic activity have also been associated with poor prognosis [3-9], suggesting that these are metastasis promoting phenotypes. The molecular biology behind these phenotypes has not been clarified, but the proliferation proteins EGFR and ERBB2, the anti-apoptosis proteins cIAP1 and BCL2, and the glucose transporter GLUT1 may be involved [10-14]. A more comprehensive characterization of the tumors is, however, needed to achieve a complete understanding of how metastatic phenotypes develop in cervical cancers.

Gene expression microarrays are useful for discovery of new genes that are regulated in metastatic tumors [2,15]. Panels of genes associated with lymph node metastasis have been identified for several cancer types by using this technique [2,16], suggesting that the gene expression program is altered in metastatic as compared to nonmetastatic tumors. Genes related to treatment outcome or lymph node metastasis have also been identified in microarray studies of cervical cancers [17-19]. Less than 20 patients were included in these studies, making it difficult to draw firm conclusions from the results. Moreover, protein expressions or copy numbers of the genes were not addressed.

In the present work we have used microarrays to identify gene expressions associated with metastatic phenotypes of cervical cancers and to investigate whether gains or losses of these genes could play a role in the transcriptional regulation and phenotype development. Locally advanced primary tumors of squamous cell origin, all receiving curative radiotherapy, were included. Treatment outcome differs considerably among these patients, emphasizing the need for identifying and exploring risk factors. We report 31 genes that differed in expression between lymph node positive and negative tumors, as diagnosed from magnetic resonance (MR) images. These genes provided a basis for further analyses and enabled us to generate hypotheses of gene functions in metastatic tumors. The importance of gains and losses for the expression of these genes were explored by including genomic microarray data in the analyses. The gene data were further related to progression free survival, since this end point is a stronger indicator of the metastatic capacity of the tumors than the diagnosed metastatic status *per se*. Unsupervised hierarchical clustering was performed to identify coregulated genes that were probably associated with the same phenotypes.

## Results

### Differentially expressed genes in node positive *versus *node negative tumors

**Figure 1:**
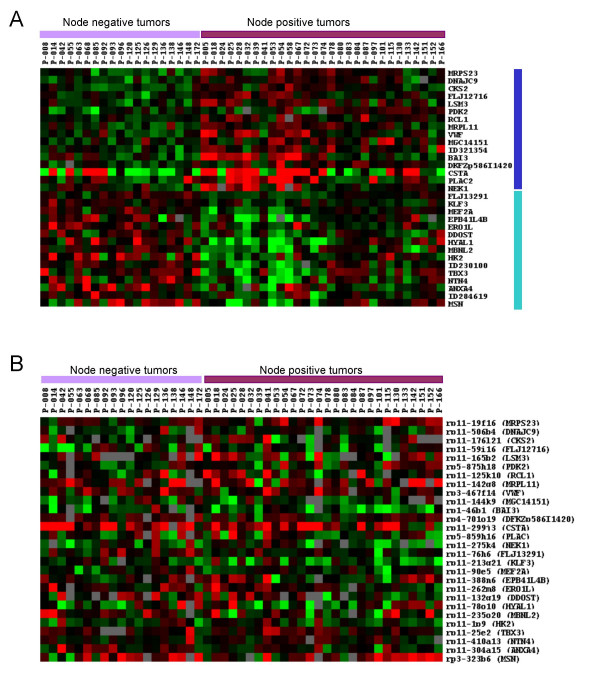
**Gene expressions and copy numbers in node negative and positive tumors**. A, Expression ratios of 31 genes that differed in expression between 19 node negative and 29 node positive cervical tumors. The data are the average log_2_ratio of two dye-swap experiments. Gene symbols (NCBI UniGene) are shown to the right. The genes indicated with a dark blue line were upregulated in node positive compared to node negative tumors, whereas those marked light blue were downregulated. The ratios were median centered, and expressions higher and lower than the median expression of that gene are shown in red and green, respectively. The most intense colors represent log_2_ratios of > 2.0 (red) and < -2.0 (green) relative to the median, whereas black represents expression close to the median value. B, Gene copy number relative to modal DNA content (log_2_ratio) of the BAC clones covering or are close to the genes in (A) for the patients presented in (A). Patient P-058 is not included, since tumor DNA was not available. BAC clone identifications and the corresponding gene symbols (NCBI UniGene) are shown to the right. Gene gains and losses are shown in red and green, respectively, whereas black represents no aberration. The most intense red and green colors represent log_2_ratios of > 0.8 and < -0.8, respectively. The log_2_ratios varied between -0.84 and 2.19.

Twenty-nine patients were diagnosed with pathologic lymph nodes in the pelvic region, including three with additional pathologic para-aortic nodes, whereas 19 patients were node negative. Thirty-one genes with major difference in expression when comparing the data of node positive and negative tumors, were selected. These were 16 genes with higher and 15 with lower expression in the node positive tumors (Figure 1A) and included genes coding for structural proteins, such as the mitochondrial ribosomal proteins MRPS23 and MRPL11, enzymes participating in metabolism, like the pyruvate dehydrogenase kinase PDK2 and the hexokinase HK2, proteins interacting with the extracellular matrix, such as moesin (MSN) and the hyaluronglucosaminidase HYAL1, the cell division cycle CDC28 protein kinase regulatory subunit 2 (CKS2), the proteinase inhibitor cystatin A (CSTA), and others with multiple or more unclear functions with regard to metastasis development, like the hypothetical proteins MGC14151, LSM3, and FLJ13291, the muscleblind-like protein MBNL2, the T-box transcription factor TBX3, the krüppel-like factor KLF3, the annexin ANXA4, and the myocyte enhancer factor MEF2A (Table 1). The data of probes representing the same gene, but with a different sequence, were always highly correlated (p < 0.0001), and the most differentially expressed probe is listed. The microarray data showed significant correlation to quantitative real time (qRT) PCR measurements of selected genes (Additional file 1).

### Gene copy numbers

**Figure 2:**
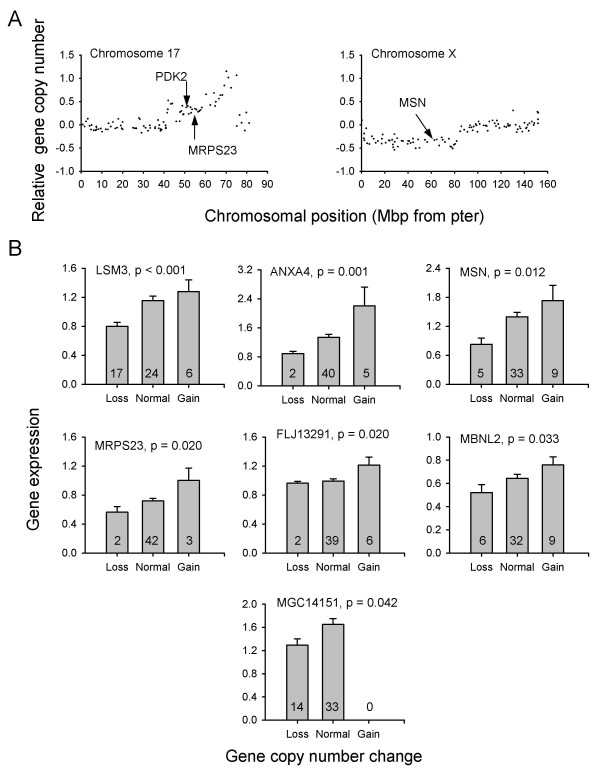
**Gene copy numbers in relation to gene expressions**. A, Gene copy number relative to model DNA content (log_2_ratio) *versus *position on chromosome 17 (left panel) and chromosome X (right panel), demonstrating gain of *PDK2 *and *MRPS23 *(chromosome 17) and loss of *MSN *(chromosome X) in two different cervical tumors. Each point represents the data for a BAC clone on the genomic microarray. For identification of gains and losses the relative copy number of neighboring clones was always considered in addition to that of the clone of interest. All amplified or deleted regions contained at least three clones. B, Gene expressions (absolute scale) in 47 tumors with loss, no aberration (normal), or gain of *LSM3, ANXA4, MSN, MRPS23, FLJ13291, MBNL2*, and *MGC14151*. These genes were the ones with a significant correlation between gene expression and copy number. The chromosomal location of the genes is listed in Table 1. *Columns*, mean values for all tumors in the groups; *bars*, standard errors. P-values for the correlations and number of tumors in each group are indicated.

Pronounced gene copy number changes were observed in most tumors, and gains on chromosome 1q, 3q, and 5p and losses on 3p were among the most frequent ones (data not shown). The 31 genes identified from the gene expression microarray analysis were selected for further analyses (Additional file 2). Gain and/or loss was seen for all these genes (Figure 1B), however, the aberrations were generally not frequent and occurred in less than half of the tumors for all but *CSTA *on 3q21. Some genes were located on the same chromosomal region (Table 1), such as *MRPS23 *and *PDK2 *on 17q (Figure 2A), and the data of these genes were highly correlated (data not shown). Significant relationship between the gene copy number changes and expressions was found for seven genes: *MRPS23, MSN*, *LSM3*, *ANXA4*, *MBNL2*, *FLJ13291*, and *MGC14151*, for which aberrations were detected in 5–23 tumors (Figure 2B). Among these genes, the copy number changes correlated with metastatic status for ANXA4 and FLJ13291 (Additional file 3). The comparison of the data between node positive and negative tumors was, however, hampered by the very few cases with aberrations.

### Protein expressions

**Figure 3:**
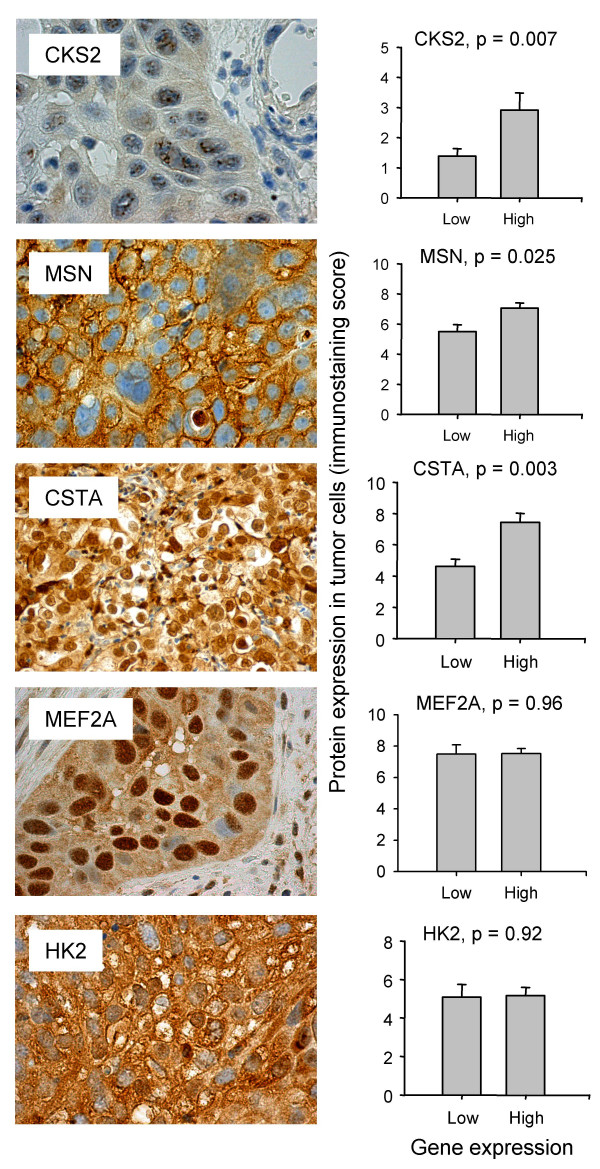
**Protein expressions in relation to gene expressions**. Immunostaining of CKS2 (nuclear), MSN (membrane/cytoplasmic), CSTA (nuclear/cytoplasmic), MEF2A (nuclear/cytoplasmic), and HK2 (cytoplasmic) in cervical tumors with high protein levels (left panels) and immunostaining score in 48 cervical tumors with low or high gene expression of *CKS2, MSN, CSTA, MEF2A*, and *HK2 *(right panels). MSN, MEF2A, and HK2 were expressed in both tumor and stroma cells, and only the immunostaining in tumor cells is included in the histograms. For CKS2 and CSTA, which were upregulated in node positive tumors, the histograms compares the immunostaining score between the quartile of tumors (n = 12) with the highest gene expression and the remaining 36 ones. For HK2, MEF2A, and MSN, which were downregulated in node positive tumors, the immunostaining score between the quartile of tumors with lowest gene expression and the remaining 36 ones is compared. *Columns*, mean values for all tumors in the groups; *bars*, standard errors. P-values for the correlations are indicated.

Immunohistochemistry was performed on five genes (*CKS2, CSTA, HK2, MEF2A, MSN*), for which differentially expression between node positive and negative tumors was observed and antibodies were commercially available, to assess the protein expression and identify the cell types expressing the gene. All proteins were expressed in tumor cells (Figure 3), and expression was also seen in stroma cells for HK2, MEF2A, and MSN. The expression levels differed considerably among the tumors for all proteins. To search for relationships between gene and protein expression, the immunostaining in both tumor and stroma cells was considered by calculating an average score for the two cell types using data on tumor cell fraction. For CSTA and MSN there was a linear relationship between protein and gene expression, but for CKS2 a certain transcript level seemed to be needed before protein was detected (data not shown). We therefore compared the immunostaining score between the quartile of cases (n = 12) with the highest gene expression and the remaining 36 ones for CKS2 and CSTA, which were upregulated in node positive tumors, and between the quartile of cases with the lowest gene expression and the remaining 36 ones for MSN, HK2, and MEF2A, which were downregulated in node positive tumors. The immunostaining score of CKS2, CSTA, and MSN was significantly higher in the cases with high gene expression compared to those with low (p < 0.007 for each protein), in concordance with the gene expression results. The difference remained significant when only the immunostaining in tumor cells was considered for MSN (Figure 3). No correlation between gene and protein expression was found for MEF2A or HK2, regardless of whether immunostaining in both cell types (data not shown) or only in tumor cells was considered (Figure 3).

### Progression free survival

**Figure 4:**
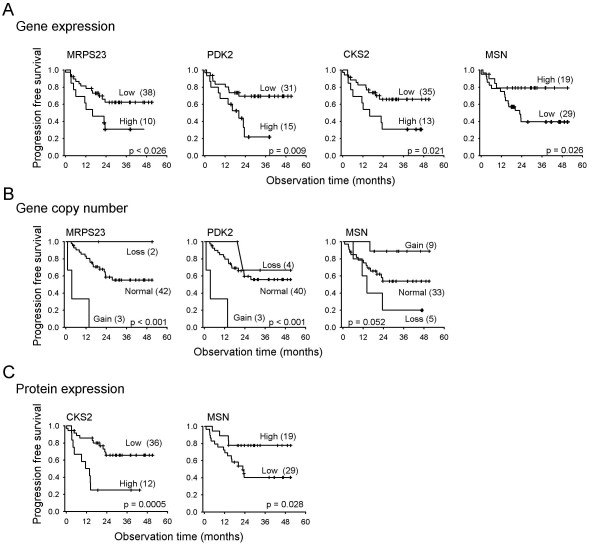
**Progression free survival analysis**. Kaplan Meier plots of progression free survival for 48 cervical cancer patients with high or low gene expression of *MRPS23, PDK2*, *CKS2*, and *MSN *(A), with loss, gain, or no change in gene copy number of *MRPS23, PDK2*, and *MSN *(B), and with high or low protein expression of *CKS2 *and *MSN *in the tumor cells (C). Number of patients in each group and p-value in log rank test are indicated. The p-values in (A) differ slightly from that in Table 2 because log-rank tests are used in (A), whereas the data in Table 2 are based on Cox regression analysis. Note the concordance in the results based on gene expressions, copy numbers, and protein expressions.

The data of the differentially expressed genes were related to progression free survival in univariate analysis to find the genes with the strongest relationship to metastasis. The correlation was significant for the expression of *MRPL11, PDK2, KLF3, MRPS23, CKS2, TBX3, LSM3*, and *MSN *(Table 2), suggesting that these genes were more strongly associated with metastatic phenotypes than the others. *MRPL11*, *CKS2*, and *PDK2 *were the most significant ones (Table 2, Figure 4A). The highest significances were generally achieved with the categorised data and are listed for all genes, although the ratios (linear or log_2_) were more significant for *MRPS23*, *CKS2, LSM3*. Tumor volume was the strongest prognostic factor among the clinical variables (Table 2). Metastastic status *per se*, as determined by MR imaging, showed no prognostic significance due to the limited number of patients, however, number of pathological lymph nodes was significant. Multivariate analysis identified *MRPL11, TBX3*, and *PDK2 *expression as independent prognostic gene variables, whereas only tumor volume was identified in a model containing the clinical variables (Table 2). Tumor volume and *PDK2 *expression remained independent prognostic variables when both clinical and gene variables were considered.

Univariate analysis of the gene copy number changes, categorised as gain, loss, or no change, showed a significant relationship to progression free survival for *MRPS23 *(*p *= 0.0002), *PDK2 *(*p *= 0.004), and *MSN *(*p *= 0.016). Although the aberrations were not frequent, occurring in 5 (*MRPS23*), 7 (*PDK2*), and 14 (*MSN*) tumors, they seemed to have a pronounced influence on the survival probability when they occurred (Figure 4B). The relationship for *MRPS23 *and *PDK2 *was similar, except for two additional losses of *PDK2*, reflecting their colocation on chromosome 17q. The three tumors with gain of *PDK2 *had a high *PDK2 *expression, however, there was no general correlation between expression and aberration for this gene (Figure 2B). The results in Figure 4B for *PDK2 *could therefore be a consequence of its colocalization with *MRPS23*. The results of *MRPS23 *and *MSN*, on the other hand, were consistent with our observations that the aberrations correlated with the expression levels of these genes (Figure 2B) and that the expression levels showed prognostic significance (Figure 4A).

In univariate analysis of the protein expressions the immunostaining score of the tumor cells was included as continuous data, without considering the score in stroma cells. The data showed a significant relationship to progression free survival for CKS2 (*p *= 0.034) and MSN (*p *= 0.025), but not for CSTA, MEF2A, and HK2. The largest difference in survival was achieved when using a cut off for the immunostaining score of 4 and 9 for CKS2 and MSN, respectively (Figure 4C). The protein data were therefore in concordance with the gene expression results, showing that high expression of *CKS2 *and low expression of *MSN *were associated with poor survival (Figure 4A).

### Gene clusters

**Figure 5:**
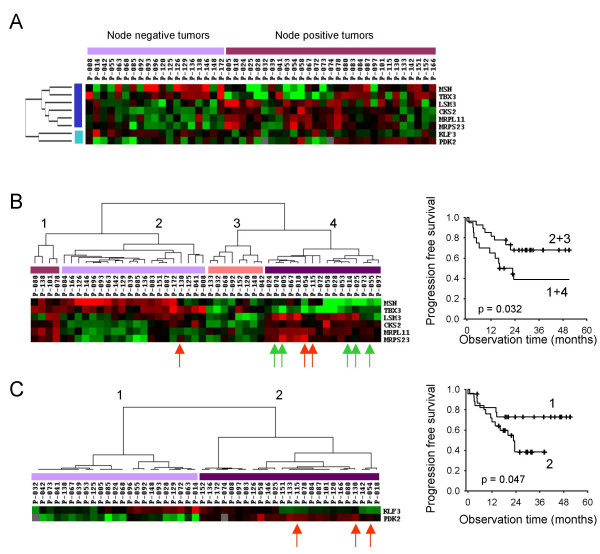
**Cluster analysis of genes and patients**. A, Cluster diagram of *MSN, TBX3, LSM3, CKS2, MRPL11, MRPS23, KLF3*, and *PDK2 *expression in 19 node negative and 29 node positive cervical tumors. B, Cluster diagram of 48 cervical tumors based on *MSN, TBX3, LSM3, CKS2, MRPL11*, and *MRPS23 *expression (left) and Kaplan Meier plots of progression free survival for patients in group 1 and 4 (high expression of *MRPL11, MRPS23, CKS2*, and *LSM3 *and low expression of *MSN *and *TBX3*) *versus *group 2 and 3 (low expression of *MRPL11, MRPS23, CKS2*, and *LSM3 *and high expression of *MSN *and *TBX3*) (right). C, Cluster diagram of 48 cervical tumors based on *KLF3 *and *PDK2 *expression (left) and Kaplan Meier plots of progression free survival for patients in group 1 (high expression of *KLF3 *and low expression of *PDK2*) *versus *groups 2 (low expression of *KLF3 *and high expression of *PDK2*) (right). Clustering was performed based on the log_2_ratios, using absolute Pearson correlation (A) or Pearson correlation (B, C) algorithms. The ratios were median centered, and expressions higher and lower than the median expression of that gene are shown in red and green, respectively. The most intense colors represent log_2_ratios of > 1.0 (red) and < -1.0 (green) relative to the median value, whereas black represents expression close to median. Arrows point to patients with gain of *MRPS23 *and *PDK2 *(red) or loss of *MSN *(green). Group numbers and p-value in log rank test are indicated in the Kaplan Meier plots.

Unsupervised clustering of the eight prognostic genes was performed based on the gene expression microarray data (log_2_ratios) to identify genes that were coregulated in the same tumors and therefore probably associated with the same phenotypes. The genes clustered into two major groups with high degree of collinearity (p < 0.05), one group with *MSN, TBX3, LSM3, CKS2*, *MRPL1*, and *MRPS23 *and another with *PDK2 *and *KLF3 *(Figure 5A), suggesting that they were associated with at least two distinct phenotypes. The data of the former group, except for *MSN *expression, showed a significant correlation to tumor volume (*p *< 0.04 for each gene), whereas no such relationship was found for *PDK2 *and *KLF3 *expression.

To ensure that the expression data of each phenotype separated the patients into groups with different survival probability, unsupervised clustering of the patients was performed. The combined expression data (log_2_ratios) of each group of coregulated genes were used in two separate analyses. Clustering based on *MSN, TBX3, LSM3, CKS2*, *MRPL1*, and *MRPS23 *expression identified four tumor groups, for which patients of group 1 and 4, with high expression of *LSM3, CKS2*, *MRPL11*, and *MRPS23 *and, in general, low expression of *MSN *and *TBX3 *compared to group 2 and 3, had the lowest survival probability (Figure 5B). Seven of the eight tumors with gain of *MRPS23 *or loss of *MSN *were in these groups. A similar analysis based on *KLF3 *and *PDK2 *expression, separated two major tumor groups, for which patients in the group with high *PDK2 *and low *KLF3 *expression had the lowest survival probability (Figure 5C). All tumors with gain of *PDK2 *were in this group. Each of the two phenotypes indicated by the coregulated genes in Figure 5A were therefore associated with poor progression free survival probability and therefore with metastasis development.

### Discussion

Genes that differed in expression between node positive and negative cervical tumors and therefore may be related to metastatic phenotypes, were identified in our study. Our data on protein expressions and gene copy numbers provided information on the cell type expressing the genes and the regulation mechanisms involved. The frequent copy number changes, especially on chromosome 1q, 3q, 3p, and 5p, were consistent with previous reports [20,21]. Copy number changes of the differentially expressed genes were, however, less common. Such changes do probably not play a role in development of the metastatic phenotypes in the majority of tumors, for which other transcriptional regulation mechanisms seem to be important. It should be noted that no general conclusion about the role of gene copy number changes in development of the metastatic phenotypes could be drawn from our study, since only selected genes were considered. Gains or losses of other genes may be important and even influence the expression of the genes addressed here.

The protein data were not correlated with the gene expressions of two of the five proteins investigated, HK2 and MEF2A. Cross-reaction of the antibodies used for immunohistochemistry to other proteins may explain this apparent discrepancy. Hence, although the MEF2A antibody used was recommended for this protein, the producer states that cross-reactions to MEF2C and MEF2D may occur to a lesser extent. The protein data may also be less representative of the entire tumor than the gene expressions, since they were derived from a single biopsy whereas several samples were used in the microarray analyses. Moreover, post-transcriptional control of the protein levels is a likely explanation of these results as well [22].

We identified two independent groups of genes with prognostic significance, suggesting the existence of at least two major metastatic phenotypes of the locally advanced stages of cervical carcinomas. None of the prognostic genes have previously been associated with metastasis in this tumor type. Genes, such as *EGFR, ERBB2, BCL2, cIAP*, and *GLUT1*, which have shown correlations to survival in protein studies [10-14], were, however, not identified here. None of these were differentially expressed between the node positive and negative tumors with the cut off used in our study and therefore not considered in the further analyses. A separate analysis showed that *EGFR *expression correlated with survival (data not shown), in concordance with previous reports [13]. The other genes may be regulated post-transcriptionally or be prognostic in larger studies. Our strategy was therefore not suited to find all prognostic genes, but ensured that the ones identified were truly associated with metastatic phenotypes. Based on the current knowledge of gene function it was possible to relate the prognostic genes to biological characteristics that have been associated with metastasis development in cervical cancers and thereby propose molecular mechanisms underlying these characteristics.

*MRPS23, MRPL11, CKS2, TBX3, LSM3*, and *MSN *constituted the largest group of coregulated and prognostic genes. The former five genes probably play a role in tumor growth, since their expression showed a significant relationship to tumor volume. *MRPS23 *and *MRPL11 *are structural components of the mitochondria. Upregulation of these genes suggests increased mitochondrial activity, energy production through oxidative phosphorylation, and a high oxygen consumption rate. *CKS2 *is involved in cell cycle control, and its activation has been associated with high proliferation of lymphoma cells and incidence of metastasis in colon carcinomas [23,24]. *LSM3 *and *TBX3 *may also participate in cell cycle control; upregulation of *LSM3 *promotes proliferation of pancreatic cancer cells, and *TBX3 *may interact with the cell cycle protein CDKN2A [25,26]. Our data are therefore consistent with increased mitochondrial activity and cellular proliferation in metastatic tumors, probably leading to rapid growth and large volumes. These phenotypes have previously been associated with poor prognosis of cervical cancer [7], and our findings point to genes that are associated with and might participate in their development. Furthermore, when gains and losses of *MRPS23 *and *LSM3 *occur, they may influence the gene expressions and possibly the development of metastatic disease.

Clustering of *MSN *together with *MRPS23, MRPL11, CKS2, TBX*, and *LSM *suggests a role of this gene in cell proliferation, consistent with previous studies [27]. Loss of MSN function may also cause cell depolarization, increased motility, and invasiveness, and therefore be directly involved in several steps during spreading [27]. *MSN *repression may therefore actively promote metastasis development in some rapidly growing tumors. Our data further suggest that gene loss leads to *MSN *downregulation and is associated with a metastatic phenotype. Loss of heterozygosity studies have shown increased imbalance on the X chromosome in lymph node metastases as compared to the primary cervical tumors, in agreement with this hypothesis [28].

The other group of coregulated and prognostic genes, *PDK2 *and *KLF3*, is probably associated with hypoxia tolerance. Activation of *PDK2 *directs glycolytically derived pyruvate towards anaerobic metabolism and lactate output rather than oxidative phosphorylation through repression of the pyruvate dehydrogenase (PDH) complex [29]. Repression of *KLF3 *may indicate increased glucose metabolism under hypoxia, although other functions involving growth, apoptosis, and angiogenesis, have also been proposed [30,31]. Our data on *PDK2 *and *KLF3 *therefore suggest activation of strategies to conserve energy and, hence, survive under hypoxia. Hypoxia and high lactate content are prognostic factors for cervical cancer [3,5,8], consistent with our hypothesis. Moreover, repression of PDH subunit alpha 1 has previously been associated with treatment outcome of this disease [17], and similar survival strategies are probably used in other tumor types, showing activation of lactate dehydrogenases [32,33]. Our data showed that tumors with concurrent gain of *PDK2 *and *MRPS23 *had high expression of *MRPS23 *and *PDK2*, large volumes above the median value, a very short survival time and, therefore, an extremely high risk for metastasis development, suggesting the combined characteristics of rapid proliferation, high oxygen consumption, and hypoxia tolerance.

The regulation of the other differentially expressed genes also contributes to the characteristics of metastatic disease. Many of them were coregulated with the prognostic ones, and could be markers of a high proliferation activity or regulated in response to hypoxia. Repression of *NTN4 *and *HYAL1 *may promote cell migration and invasive growth, whereas *BAI3, VWF*, and *EPB41L4B *probably participate in angiogenesis, attachment of tumor cells to endothelial surfaces, or reflect vascular structures in the tumors [34-36]. Moreover, regulation of *NEK1*, *CSTA*, *ANXA4*, and *DDOST *indicates activation of DNA damage repair (*NEK1*) and resistance to apoptosis (*CSTA *and possibly *ANXA4 *and *DDOST*) [37-39], whereas transcriptional downregulation of *HK2 *may be a result of glucose deprivation [22]. The roles of *MBNL2, RCL1, MGC14151, ERO1L*, *DNAJC9*, *PLAC2*, and *MEF2A *are more unclear, but may involve regulation of proteins like the insulin receptor (*MBNL2*) [40] or proteins participating in development (*MEF2A*). Elevated VWF plasma levels have been found in node positive cervical cancer patients [41], consistent with our results. Moreover, increased CSTA protein expression has been correlated with poor prognosis of breast carcinoma, whereas repression of *ERO1L *has been shown to increase the recurrence probability of pulmonary adenocarcinoma [42,43], suggesting that these genes are related to metastasis development also in other tumor types.

## Conclusion

We have identified genes associated with major metastatic phenotypes of cervical cancers that probably are related to rapid proliferation and hypoxia. Copy number changes of the genes may be involved in development of these phenotypes in some cases, but other mechanisms for transcriptional regulation are probably important in the majority of tumors. The gene expressions may be useful markers of cancer spread, since they showed a stronger relationship to progression free survival than the metastatic status and number of pathological lymph nodes and added prognostic information to tumor volume. Although our findings need to be confirmed in larger studies with more patients, they provide a useful basis for further investigations to reveal the mechanisms underlying development of these phenotypes.

## Methods

### Patients, lymph node involvement, and treatment

Forty-eight patients with primary squamous cell carcinoma of the uterine cervix, diagnosed in the period 2001 – 2004 at Health Enterprise Rikshospitalet-Radiumhospitalet, were included. Tumor stage (The Fédération Internationale des Gynaecologistes et Obstetristes) was 2a (1 patient), 2b (29), 3b (14), and 4a (4), and tumor grade was 2 or 3. Lymph nodes were evaluated at the time of diagnosis with MR imaging, using axial fast spin echo T_2_-weighted images. A pelvic phased array coil was used for nodes below the promontory. The body coil was used for retroperitoneal nodes from the promontory to the upper pole of the kidneys. Lymph nodes were classified as pathologic when the minimal axial diameter exceeded 10 mm in oval nodes or 8 mm in round nodes. Using these criteria for metastasis in pelvic lymph nodes in prostatic and urinary bladder carcinoma, the sensitivity and specificity were 75% and 98%, respectively [44]. Some tumors may therefore falsely be classified as node negative. Pretreatment tumor volume, determined from the MR images as π/6·*x*·*y*·*z*, where *x *is largest diameter in the sagittal plane through the cervix length axis and *y *and *z *are diameters in a plane orthogonal to this axis, ranged from 17 – 336 cm^3 ^(median 46 cm^3^).

All patients received external irradiation and brachytherapy with curative intent. External irradiation, 50 Gy to tumor and parametria and 45 Gy to the rest of the pelvic region, was delivered by use of a linear accelerator in 25 fractions five times per week. A ^192^Ir stepping source was used for endocavitary brachytherapy, employing 21Gy in five fractions to point A. Adjuvant cisplatin (40 mg/m^2^) was given weekly in maximum 6 courses during the period of external radiation. Twenty-two patients completed all cisplatin courses, whereas the others had dose reduction or delay due to toxicity problems. The follow up included clinical examinations every 3^rd ^month for the first 2 years, twice a year the next three years, and thereafter once a year. MR imaging of pelvis and retroperitoneum and X-ray of thorax were performed when symptoms of recurrent disease were seen. Progression free survival, defined as the time between diagnosis and the first event of locoregional and/or distant relapse or cancer related death, was used as end point. Four patients, who died due to cerebral hemorrhage or treatment related complications, were censored. All other deaths were cancer related. Observation time, calculated for the patients that were still alive, ranged from 15 – 51 months (median 36 months). The study was approved by the regional committee of medical research ethics in southern Norway (REK no. S-01129), and written informed-consent was achieved from all patients.

### Tumor specimens

One – four biopsies (median 3 biopsies), approximately 5 × 5 × 5 mm in size, were used in the microarray analyses of each tumor, minimizing confounding effects caused by intratumor heterogeneity in gene expressions and copy numbers [21]. The biopsies were taken from different locations of the tumor at the time of diagnosis, immediately snap-frozen in liquid nitrogen, and stored at -80°C. All biopsies had more than 50% tumor cells in hematoxylin and eosin stained sections, derived from the central part of the specimen. Median tumor cell fraction was 70% (range 50–90%) for both the node positive and negative tumor groups. Total RNA was isolated by use of Trizol reagent (Invitrogen, Carlsbad, CA) followed by double precipitation with isopropanol and final precipitation with 5 M lithium chloride [45]. Genomic DNA was isolated according to a standard protocol, including proteinase K, phenol, chloroform, and isoamylalcohol [46]. A separate biopsy was fixed in neutral 4% buffered formalin and used for immunohistochemistry.

### Gene expression microarray analysis

Gene expressions were studied using array slides produced at the Microarray Facility at Health Enterprise Rikshospitalet-Radiumhospitalet, containing 15000 cDNA clones. Labeled cDNA was synthesized from 20 μg total RNA by anchored oligo(dT)-primed reverse transcription, using SuperScript II reverse transcriptase (Invitrogen) and Fairplay labeling kit (Stratagene, La Jolla, CA) in the presence of either Cy3-dUTP or Cy5-dUTP (Amersham Pharmacia Biotech Inch., Piscataway, NJ). The labeled cDNA of each tumor was co-hybridized with that of a reference sample (Universal Human Reference RNA, Stratagene) to the array slides overnight at 65°C, using an automated hybridization station (GeneTAC, Genomic Solutions/Perkin Elmer, Boston, MA). All hybridizations were performed twice in a dye-swap design. Scanning and image analysis were performed with an Agilent scanner (Agilent Technologies Inc., Palo Alto, CA) and the GenePix 4.1 image analysis software (Axon Instruments Inc., Union City, CA), respectively. Data preprosessing included correction of saturated intensities [47], filtering of weak and bad spots, and lowess normalization. Our protocol for the cDNA microarray experiments has revealed reliable results in agreement with northern analyses in previous studies on cell lines [45].

### Quantitative real time PCR

For validation of the microarray data we performed qRT PCR of four genes (*HK2, KLF3, MRPS23, PDK2*) in all 48 tumors and two genes (*CSTA, DDOST*) in twelve tumors. Pre-designed, gene-specific TaqMan probe and primer sets (Applied Biosystems, Foster City, CA) and a 7500 Sequence Detector (Perkin-Elmer) were used (Additional file 4). Ten ng cDNA, which was synthesized from total RNA by use of Superscript II transcriptase (Invitrogen), was employed. Conditions for amplification were one cycle of 95°C in 10 min followed by 40 cycles of 95°C in 15 sec and 60°C in 1 min. The reactions were carried out in triplicate in a 25 μl reaction volume and a 96-well plate format. Gene expression was calculated using the delta-delta method, were the *B2M *gene served as an internal control and the reference sample used in the microarray experiments served as a calibrator [48].

### Genomic microarray analysis

Gains and losses of the genes identified in the cDNA microarray analysis were assessed using genomic array slides produced by the Microarray Facility at Health Enterprise Rikshospitalet-Radiumhospitalet, containing 5000 unique BAC clones (RPCI-11 library, Wellcome Trust Sanger Institute, Cambridge, UK) that covered the whole genome with a resolution of approximately 1 Mbp. The BAC clone localizations were obtained from Ensembl [49]. The clone identifications corresponding to the genes of interest are listed in Additional file 2. Forty-seven of the 48 tumors were included, for which genomic DNA was available. One half μg of digested and ethanol-purified DNA and normal female reference DNA were labeled by a random primer reaction with Cy3-dUTP and Cy5-dUTP (Amersham Pharmacia Biotech Inch.), respectively, and co-hybridized to the array slides in 48 hours at 37°C by use of an automated hybridization station (GeneTAC, Genomic Solutions/Perkin Elmer) and our established protocol [50]. Scanning, image analysis, and normalization were performed as described previously, using an Agilent scanner (Agilent Technologies Inc.), the GenePix 4.1 image analysis software (Axon Instruments Inc.), and the M-CGH data preprosessing software, respectively [50]. Gene copy number changes were determined from the log_2_ratios by using a cut off value specific for each tumor, taking into account the tumor cell fraction, the DNA ploidy, and the empirically determined dynamics of the experiments (Figure 2A). This procedure provides results in agreement with fluorescence-in situ-hybridization data [50,51]. The data were classified as gain, loss, or no change in copy number relative to the modal DNA content.

### Immunohistochemistry

Commercially available antibodies for CKS2, CSTA, HK2, MEF2A, and MSN were used. Paraffin tissue sections of all tumors were stained using the Dako EnVision™ + System, Peroxidase (DAB) (K4007 and K4011, Dako Corporation, CA) and Dakoautostainer. Monoclonal antibodies MSN (clone 38/87, Sigma, MI), CSTA (clone WR-23/2/3/3, Sigma), CKS2 (clone 1F7G5, Zymed Laboratories Inc., CA), and polyclonal antibodies HK2 (AB3279, Chemicon International, CA) and MEF2A (C-21, Santa Cruz Biotechnology, CA) were used. Detailed information on the immunohistochemical staining has been published previously [52]. All series included positive and negative controls that gave satisfactory results. Negative controls included substitution of the monoclonal antibody with mouse myeloma protein of the same subclass and concentration as the monoclonal antibody and substitution of the polyclonal primary antibody with normal rabbit IgG of the same concentration as the polyclonal antibody. Immunostaining was scored on a 3-tiered scale for both intensity (absent/weak, 1; moderate, 2; strong, 3) and extent of staining (percentage of positive tumor cells < 10%, 1; 10–50%, 2; > 50%, 3). The scoring results of intensity and extent were multiplied to give a composite score ranging from 1 to 9 for each tumor.

### Statistical analysis

The SAM (Significance Analysis of Microarrays) program [53] with a false discovery rate of 10% was applied on the log_2_ratios to find the genes that differed most in expression between the node positive and negative tumors. This method does not take into account the increased information and certainty in the data achieved from dye-swap designs with two experiments on each tumor. The analysis was therefore performed on the raw data sets. The differentially expressed genes were selected and the average expression ratios were calculated from the log_2_ratios of the two dye-swap experiments for further use.

The data of the differentially expressed genes were included in Cox univariate and multivariate analysis of progression free survival, using the SPSS software. When the variables are semi continuous, the covariate values may be any uniform function of these. We tested three functions for the gene expression data: the log_2_ratio, the ratio, and the categorized form, and genes showing significance (*p *< 0.05) for one of these functions were identified as major metastasis genes. In the analysis of categorized data, the tumors were divided into three groups; one group of 12 tumors with the highest expression ratios, another group of 24 tumors with intermediate ratios, and a third group of 12 tumors with the lowest ratios. The survival probability of the three groups were compared in Kaplan Meier plots to ensure that there was a monotonous increase or decrease in survival among the groups. The categorized form was chosen as approximately the group of 12 tumors *versus *the others. Unsupervised hierarchical clustering of the gene expression data was performed based on the log_2_ratios, using a program developed for this purpose [54].

### Array express accession

The raw data and the processed data file from the cDNA microarray platform have been deposited to the Array Express repository (E-TABM-146).

## Authors' contributions

HL conceived and designed the study, analyzed data, and wrote the article. RSB carried out the genomic microarray experiments and participated in data analysis. DHB performed the gene expression microarray and PCR experiments. RH performed the immunohistochemistry and analysis of the histological sections. OK contributed to the statistical analyses and helped to draft the manuscript. KK carried out MR investigations and evaluated the MR images. HO and KS contributed with clinical samples and discussions. GBK was responsible for the clinical protocol, contributed to the design of the study, and provided clinical samples and data. TS contributed to the design of the study and helped to draft the manuscript. All authors read and approved the final manuscript.

## Supplementary Material

Additional file 1 Validation of gene expression microarray data with qRT PCR. Click here for file

Additional file 2 BAC clone identification of the differentially expressed genes. Click here for file

Additional file 3 Gene copy number change in relation to metastatic status. Click here for file

Additional file 4 TaqMan gene expression assays for qRT PCR. Click here for file

## Figures and Tables

**Table 1:** Genes differentially expressed in node positive versus node negative cervical tumors

**Reporter ID^1^**	**Gene symbol^2^**	**Cytoband**	**Description^2^**	**Ratio^3^**	**Function^4^**
Upregulated in node positive tumors					
32134	DNAJC9	10q22.2	DnaJ (Hsp(40) homolog, subfamily C, member 9	1.35	Protein folding
824723	MRPS23	17q22	Mitochondrial ribosomal protein S23	1.33	Structural component of mitochondrial ribosome
144880	PLAC2	19p13.3	Placenta-specific 2	1.47	Unknown
840486	VWF	12p13.3	Von Willebrand factor	1.35	Coagulation
345957	CSTA	3q21	Cystatin A	2.24	Lysosomal proteinase inhibition
49860	PDK2	17q21.33	Pyruvate dehydrogenase kinase, isoenzyme 2	1.23	Anaerobe metabolism
359119	CKS2	9q22	CDC28 protein kinase regulatory subunit 2	1.25	Cell cycle regulation
321354			In multiple clusters	1.27	
825012	MGC14151	17p13.1	Hypothetical protein MGC14151	1.29	
796176	LSM3	3p25.1	Hypothetical protein LOC285378	1.31	MRNA splicing
825229	MRPL11	11q13.3	Mitochondrial ribosomal protein L11	1.24	Structural component of mitochondrial ribosome
50671	FLJ12716	4q35.1	FLJ12716 protein	1.24	
50491	BAI3	6q12	Brain-specific angiogenesis inhibitor 3	1.37	Angiogenesis
129516	RCL1	9p24.1	RNA terminal phosphate cyclase-like 1	1.21	Maturation of 18S RNA
290607	NEK1	4q33	NIMA (never in mitosis gene a) – related kinase 1	1.32	DNA damage repair
128208	DKFZp586I1420	7p15.1	Hypothetical protein DKFZp586I1420	1.21	
		
Downregulated in node positive tumors					
210698	EPB41L4B	9q31	Erythrocyte membrane protein band 4.1 like 4B	0.71	Structural constituent of cytoskeleton
810391	HYAL1	3p21.3	Hyaluronglucosaminidase 1	0.62	Degradation of extracellular matrix components
1637282	HK2	2p13	Hexokinase 2	0.70	Glucose metabolism
320392	MBNL2	13q32.1	Muscleblind-like 2 (Drosophila)	0.70	MRNA splicing
767769	FLJ13291	16q22.1	Hypothetical protein FLJ13291	0.82	
126519	KLF3	4p14	Krüppel-like factor 3 (basic)	0.83	Glucose metabolism, growth, apoptosis, angiogenesis
230100			In multiple clusters	0.76	
347546	ERO1L	14q22.1	ERO1-like (S. cerevisiae)	0.76	Oxidative protein folding
131362	MSN	Xq11.2	Moesin	0.71	Cell-cell and -extracellular matrix interaction
137456	TBX3	12q24.1	T-box 3 (ulnar mammary syndrome)	0.74	Regulation of growth and developmental processes
755506	ANXA4	2p13	Annexin A4	0.72	Growth regulation, apoptosis, calcium signaling
284619			In multiple clusters	0.81	
143661	NTN4	12q22	Netrin 4	0.71	Cell-cell and -extracellular matrix interaction
713469	MEF2A	15q26	Myocyte enhancer factor 2A	0.79	Growth factor related transcription, differentiation
251135	DDOST	1p36.1	Dolichyl-diphosphooligosaccharide-protein glycosyltransferase	0.72	Glycosylation, apoptosis

^1^All genes have been sequence verified. They are listed according to their score in SAM analysis.

^2^Gene symbol and description are derived from the NCBI UniGene database [55].

^3^Expression ratio refers to node positive versus negative tumors.

^4^Function is derived from the SOURCE database [56] and published literature.

**Table 2:** Cox regression analysis of progression free survival

	**Univariate analysis**			**Multivariate analysis**		
**Covariate^1^**	**p**	**Relative risk**	**95% CI**	**p**	**Relative risk**	**95% CI**
Model containing clinical variables alone						
Tumor volume	< 0.0001	1.01	1.01 – 1.02	< 0.0001	1.01	1.01 – 1.02
Number of pathological lymph nodes	0.015	1.3	1.1 – 1.7	0.24		
Metastatic status	0.30	1.7	0.64 – 4.3	0.47		
						
Model containing gene variables alone						
Gene expressions						
MRPL11	0.0001	5.6	2.3 – 14	0.001	4.9	1.9 – 13
PDK2	0.013	3.2	1.3 – 7.9	0.008	3.8	1.4 – 10
CKS2	0.026	2.7	1.1 – 6.6	0.30		
TBX3	0.028	0.37	0.15 – 0.90	0.006	0.24	0.09 – 0.67
MRPS23	0.032	2.7	1.1 – 6.9	0.87		
MSN	0.035	0.31	0.10 – 0.92	0.40		
KLF3	0.035	0.37	0.15 – 0.93	0.47		
LSM3^2^	0.058	2.7	0.97 – 7.4	0.30		
						
Model containing clinical and gene variables						
Tumor volume				< 0.0001	1.01	1.01 – 1.02
PDK2				0.013	3.3	1.3 – 8.3
MRPL11				0.26		
TBX3				0.12		

^1^Categorized variables were used for the gene variables and metastatic status, whereas the linear form were used for tumor volume and number of pathological lymph nodes.

^2^*LSM3 *expression did not reach a p-value below 0.05 when categorized data were used, but is included among the prognostic genes since analysis based on log_2_ratios showed a p-value of 0.044.
