# Supplementary material for: Gene expressions and copy numbers associated with metastatic phenotypes of uterine cervical cancer
Source: BMC Genomics. 2006 Oct 20;7:268. doi: 10.1186/1471-2164-7-268 (PMC1626467; doi:10.1186/1471-2164-7-268)
Supplement: Additional file 1 — Validation of gene expression microarray data with qRT PCR. [file 1471-2164-7-268-S1.doc]

**Figure A1 – Validation of gene expression microarray data with qRT PCR.**

Gene expressions (log_2_ratio) by cDNA microarrays *versus* the corresponding qRT PCR data for *CSTA, DDOST, HK2, KLF3, MRPS23,* and *PDK2* of 12 (*CSTA, DDOST*) or 48 (*HK2, KLF3, MRPS23, PDK2*) cervical tumors. *Points*, the average data from a dye-swap microarray experiment or three PCR experiments of a single tumor. Duplicate points are shown for *CSTA*, for which two probes on the microarray represented the gene. Note that consistent results were achieved with the two techniques, but there was a systematic shift in the data when different genes were compared probably caused by different PCR efficiency of the primers. Pearson Product Moment correlation coefficient and p-value for the entire data set are indicated. The p-values for the individual genes were 0.0073 (*CSTA*), 0.038 (*CSTA*), 0.78 (*DDOST*), 0.012 (*HK2*), 0.088 (*KLF3*), 0.0002 (*MRPS23*), and 0.014 (*PDK2*).
