# Supplementary material for: Gene expressions and copy numbers associated with metastatic phenotypes of uterine cervical cancer
Source: BMC Genomics. 2006 Oct 20;7:268. doi: 10.1186/1471-2164-7-268 (PMC1626467; doi:10.1186/1471-2164-7-268)
Supplement: Additional file 2 — BAC clone identification of the differentially expressed genes. [file 1471-2164-7-268-S2.doc]

| **Table A1. Clone identification of the differentially expressed genes** | | | |
| --- | --- | --- | --- |
| Gene symbol | **Cytoband** | **BAC clone ID**^1^ |  |
| DNAJC9 | 10q22.2 | RP11-506B4 |  |
| MRPS23 | 17q22 | RP11-19F16 |  |
| PLAC2 | 19p13.3 | RP5-859H16 |  |
| VWF | 12p13.3 | RP3-467F14 |  |
| CSTA | 3q21 | RP11-299J3 |  |
| PDK2 | 17q21.33 | RP5-875H18 |  |
| CKS2 | 9q22 | RP11-176L21 |  |
| MGC14151 | 17p13.1 | RP11-144K9 |  |
| LSM3 | 3p25.1 | RP11-165B2 |  |
| MRPL11 | 11q13.3 | RP11-142G8 |  |
| FLJ12716 | 4q35.1 | RP11-59I16 |  |
| BAI3 | 6q12 | RP1-46B1 |  |
| RCL1 | 9p24.1 | RP11-125K10 |  |
| NEK1 | 4q33 | RP11-275K4 | |
| DKFZp586I1420 | 7p15.1 | RP4-701O19 | |
| EPB41L4B | 9q31 | RP11-388N6 | |
| HYAL1 | 3p21.3 | RP11-78O10 | |
| HK2 | 2p13 | RP11-1P9 | |
| MBNL2 | 13q32.1 | RP11-235O20 | |
| FLJ13291 | 16q22.1 | RP11-76H6 | |
| KLF3 | 4p14 | RP11-213G21 | |
| ERO1L | 14q22.1 | RP11-262M8 | |
| MSN | Xq11.2 | RP3-323B6 | |
| TBX3 | 12q24.1 | RP11-25E2 | |
| ANXA4 | 2p13 | RP11-304A15 | |
| NTN4 | 12q22 | RP11-410A13 | |
| MEF2A | 15q26 | RP11-90E5 | |
| DDOST | 1p36.1 | RP11-132G19 | |
| ^1^The clone covering or being close to the gene was used. There were no clones for the gene expression reporter IDs 321354, 230100, and 284619, which were found in multiple clusters. | | | |
