# Supplementary material for: Gene expressions and copy numbers associated with metastatic phenotypes of uterine cervical cancer
Source: BMC Genomics. 2006 Oct 20;7:268. doi: 10.1186/1471-2164-7-268 (PMC1626467; doi:10.1186/1471-2164-7-268)
Supplement: Additional file 3 — Gene copy number change in relation to metastatic status. [file 1471-2164-7-268-S3.doc]

**Figure A2 – Gene copy number change in relation to metastatic status.**

Gene copy number change of *ANXA4* (A) and *FLJ13291* (B) in node negative (n = 19) and node positive (n = 29) tumors. *ANXA4* and *FLJ13291* were those among the 31 differentially expressed ones with a significant correlation between gene copy number change and metastatic status. The gene copy number of each tumor was classified as gain, loss, or no change relative to the modal DNA content and assigned the values 1, -1, and 0, respectively. *Columns*, mean values for all tumors in the group; *bars*, standard errors. P-values for the correlations are indicated.
