# Supplementary material for: Gene expressions and copy numbers associated with metastatic phenotypes of uterine cervical cancer
Source: BMC Genomics. 2006 Oct 20;7:268. doi: 10.1186/1471-2164-7-268 (PMC1626467; doi:10.1186/1471-2164-7-268)
Supplement: Additional file 4 — TaqMan gene expression assays for qRT PCR. [file 1471-2164-7-268-S4.doc]

| Table A2. TaqMan gene expression assays for qRT PCR | |
| --- | --- |
| **Gene symbol** | **TaqMan assay^1^** |
| MRPS23 | Hs00608544_m1 |
| PDK2 | Hs00176865_m1 |
| KLF3 | Hs00610885_m1 |
| HK2 | Hs00606086_m1 |
| CSTA | Hs00193257_m1 |
| DDOST | Hs00193263_m1 |
| B2M | 4326319E |
| ^1^Assays were from Applied Biosystems, Foster City, CA. | |
